# MolViewStories: Interactive molecular storytelling

**DOI:** 10.1002/pro.70540

**Published:** 2026-03-19

**Authors:** Terézia Slanináková, Zachary Charlop‐Powers, Viktoriia Doshchenko, Alexander S. Rose, Adam Midlik, Anna Sekuła, Neli Fonseca, Kyle L. Morris, Stephen K. Burley, Sameer Velankar, Jennifer Fleming, Brinda Vallat, Ludovic Autin, David Sehnal

**Affiliations:** ^1^ National Centre for Biomolecular Research, Faculty of Science Masaryk University Brno Czechia; ^2^ Institute of Computer Science Masaryk University Brno Czechia; ^3^ Independent; ^4^ Central European Institute of Technology Masaryk University Brno Czechia; ^5^ Protein Data Bank in Europe European Molecular Biology Laboratory's European Bioinformatics Institute Cambridgeshire UK; ^6^ Electron Microscopy Data Bank, European Molecular Biology Laboratory's European Bioinformatics Institute Cambridgeshire UK; ^7^ Research Collaboratory for Structural Bioinformatics Protein Data Bank and the Institute for Quantitative Biomedicine, Rutgers The State University of New Jersey Piscataway New Jersey USA; ^8^ Research Collaboratory for Structural Bioinformatics Protein Data Bank, San Diego Supercomputer Center University of California La Jolla California USA; ^9^ Cancer Institute of New Jersey, Rutgers The State University of New Jersey New Brunswick New Jersey USA; ^10^ Department of Chemistry and Chemical Biology, Rutgers The State University of New Jersey Piscataway New Jersey USA; ^11^ Rutgers Artificial Intelligence and Data Science Collaboratory, Rutgers The State University of New Jersey Piscataway New Jersey USA; ^12^ Department of Integrative Structural and Computational Biology The Scripps Research Institute La Jolla California USA

**Keywords:** interactive molecular visualization, Mol* Viewer, molecular storytelling, MolViewSpec, open‐source software, PDB, reproducible visualization, structural biology, web‐based tools

## Abstract

Effectively communicating knowledge related to molecular structures and their associated data remains a challenge, as traditional static figures limit interactivity and professional visualization tools often require substantial expertise. MolViewStories addresses these limitations by providing an open‐source, web‐based platform for creating and sharing interactive, narrative‐driven molecular visualizations. The platform uses the MolViewSpec standard for reproducible scene specification, extended to support animations, interactive descriptions, and synchronized audio commentary. Visualization is powered by the Mol* Viewer, which leverages Web Graphics Library (WebGL) for efficient 3D rendering and WebXR for immersive virtual and augmented reality experiences. Users can construct molecular narratives through an intuitive graphical interface or a command‐line workflow, enabling both exploratory and automated use. Completed stories can be shared online, exported locally, or distributed as self‐contained packages that remain functional indefinitely. Each story is assigned a persistent uniform resource locator (URL) and can be modified or reused as a template, promoting collaboration and community‐driven content creation. We demonstrate the capabilities of MolViewStories through a diverse set of narratives illustrating its broad applicability to research communication, education, and public outreach. Together, these examples highlight how interactive, web‐based storytelling can make molecular data more accessible, reproducible, and engaging. MolViewStories is freely available at https://molstar.org/mol-view-stories with open‐source code accessible at https://github.com/molstar/mol-view-stories.

## 1 INTRODUCTION

Macromolecular structure data have expanded dramatically in recent years. As of October 2025, the Protein Data Bank (PDB) archives over 240 thousand experimentally determined structures (wwPDB consortium, 2019), providing atomic‐level insight into biomolecules across all domains of life. Complementary experimental archives, such as the Electron Microscopy Data Bank (EMDB) (The wwPDB Consortium, 2024) and the Electron Microscopy Public Image Archive (EMPIAR) (Iudin et al., 2023), have further broadened the landscape by providing access to three‐dimensional density maps and raw image data from cryo‐electron microscopy and tomography. Together, these resources enable integrative structural biology at multiple resolutions, from atomic models to cellular environments. Computational resources further extend this coverage: the AlphaFold Protein Structure Database (AFDB) now provides over 214 million predicted models (Fleming et al., 2025), the SWISS‐MODEL Repository hosts approximately 3.75 million homology‐based models (Kiefer et al., 2009), and ModelArchive contains more than 600,000 deposited computational structures (Tauriello et al., 2025). Community initiatives such as ColabFold DB (Mirdita et al., 2022) and the 3D‐Beacons Network (Fleming et al., 2025; Varadi, Nair, et al., 2022) further integrate experimental and predicted models, bringing three‐dimensional structural information to nearly the entire known protein‐sequence space.

Despite this unprecedented access, effectively interpreting and communicating structural data remain significant challenges. Established open‐source tools such as Mol* (Sehnal et al., 2021), Jmol (http://www.jmol.org/) (Herráez, 2006), ChimeraX (Pettersen et al., 2021), and PyMOL (Rosignoli & Paiardini, 2022) provide powerful environments for structural analysis, while animation platforms including YASARA Dynamics (Land & Svedendahl Humble, 2018), eMovie (Hodis et al., 2007), mMaya (Riggi et al., 2024), ePMV (Johnson et al., 2011), and Molecular Nodes (Johnston et al., 2025) link molecular data to professional 3D rendering workflows. Web‐based tools such as PolyviewMM (Porollo & Meller, 2010), MovieMaker (Maiti et al., 2005), PMG (Autin & Tufféry, 2007), activeICM (Raush et al., 2009), and Michelanglo (Ferla et al., 2020) automate figure and animation generation, and educational resources like Proteopedia (Hodis et al., 2008), FirstGlance in Jmol (https://bioinformatics.org/firstglance), and Jolecule (https://jolecule.com/) integrate interactive 3D models with explanatory text.

Molecular illustrations, exemplified by David S. Goodsell's watercolors and Drew Berry's animations, have demonstrated the communicative power of narrative‐driven visualization. Building on these artistic and conceptual traditions, tools such as cellVIEW (Le Muzic et al., 2015), CellWalk (https://cellwalk.ca/) and Mesoscale Explorer (Rose et al., 2024) introduced guided, interactive tours for exploring large biological assemblies.

Despite recent advances, a gap remains for a web‐native, low‐barrier system that combines rigorous structural analysis with reproducible, narrative‐driven visualization and effortless sharing. To fill this gap, we present MolViewStories, a browser‐based platform for authoring molecular narratives that integrate interactive 3D visualization, interpolated animation, and optional audio narration. Built on the Mol* (Midlik et al., 2025) and MolViewSpec (Bittrich et al., 2024) frameworks, it combines the graphical power of Mol* with the flexibility and reproducibility of MolViewSpec. Backed by an active open‐source community, it is designed for long‐term continuity and growth. MolViewStories transforms complex structural data into accessible, engaging narratives, advancing the communication of molecular science in an era of data abundance.

The following sections describe the design and realization of MolViewStories, outlining its key features, software architecture, and implementation. Together, these components establish an open and extensible platform for creating, managing, and sharing interactive molecular narratives through modern web technologies. Building on this foundation, the Results and Discussion section demonstrates the versatility of MolViewStories across a range of example stories that highlight its applicability to research communication, education, and outreach.

## 2 METHODS

MolViewStories brings together modern web technologies and established molecular visualization frameworks. The goal is to provide an accessible, reproducible, and interactive platform for molecular storytelling. This section first summarizes the key functional features of the system. It then describes the architecture and implementation that enable efficient authoring, rendering, and sharing of molecular narratives.

### 2.1 Features

MolViewStories provides a comprehensive framework for creating, customizing, and sharing interactive, narrative‐driven molecular visualizations. Its design emphasizes accessibility, reproducibility, and flexibility, enabling both novice and expert users to construct detailed molecular narratives without specialized software. By combining MolViewSpec, a declarative scene specification model, with modern web technologies, the platform allows molecular visualizations to be rendered and explored interactively in any browser with high reproducibility. The key features of MolViewStories are summarized below.

*Interactive storytelling*. The construction of narrative‐driven molecular visualizations is enabled through scene sequences with animated transitions and optional audio commentary, facilitating clear illustration of concepts such as conformational changes, binding events, and molecular mechanisms. By framing visualizations as stories, MolViewStories enhances understanding and engagement, guiding users through molecular processes in a coherent and memorable way.

*Code‐driven scene builder*. Scenes are defined using MolViewSpec format, extended with animation support for time‐dependent transitions. This format ensures reproducibility, automation, and fine‐grained control of visualizations.

**FIGURE 1:**
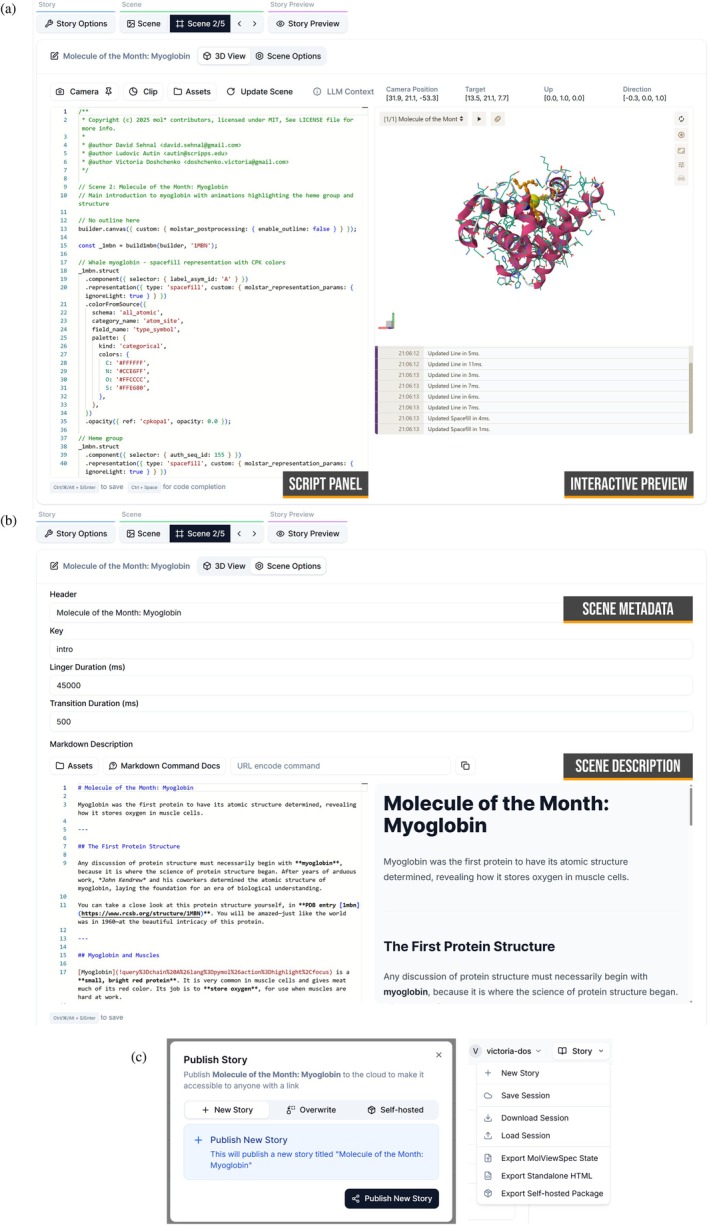
MolViewStories user interface. (a) Scene builder showing the integrated script panel and live 3D preview for interactive scene composition. (b) Scene metadata and description editor supporting Markdown formatting for rich text, hyperlinks, and embedded media such as audio narration. (c) Sharing and export options allowing users to publish stories to the cloud (and generate persistent URLs), or export self‐contained offline packages for long‐term accessibility.

*Modern user interface*. The application provides two complementary user interfaces tailored to different workflows. The web‐based interface (see Figure 1) allows users to interactively script, preview, and annotate molecular stories directly in the browser, offering a powerful environment for exploratory and educational use. In parallel, the command‐line interface (CLI) supports scripting, automation, and integration with computational pipelines, enabling advanced users to generate and manage stories programmatically.

**FIGURE 2:**
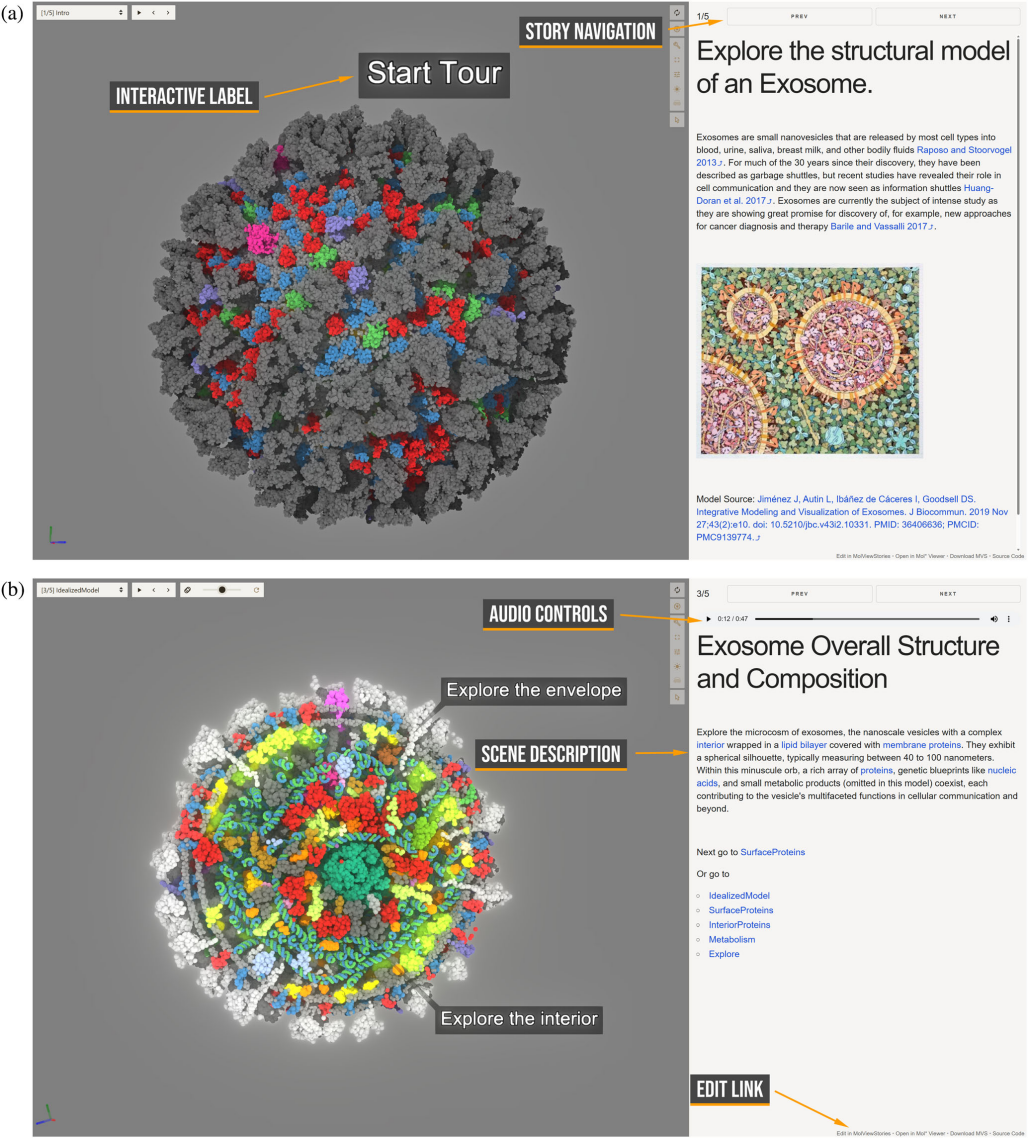
Story Viewer interface. The interface presents interactive 3D molecular scenes accompanied by a scene description, and, if provided by the author of the story, audio narration. The user can edit the story by opening it in the MolViewStories app. (a) Initial view with interactive scene elements for navigation, (b) subsequent scene illustrating specific concepts about the structure. The figure showcases the Exosome story described in the Results and Discussion section.

*Lightweight story viewer*. MolViewStories includes a standalone viewer application based on the Mol* Viewer for presenting completed stories. The story viewer is optimized for performance and simplicity, requiring no additional dependencies and can be easily integrated into third‐party platforms or web‐based resources. This flexibility enables seamless incorporation of interactive molecular narratives into online publications, educational materials, or institutional databases (see Figure 2).

**FIGURE 3:**
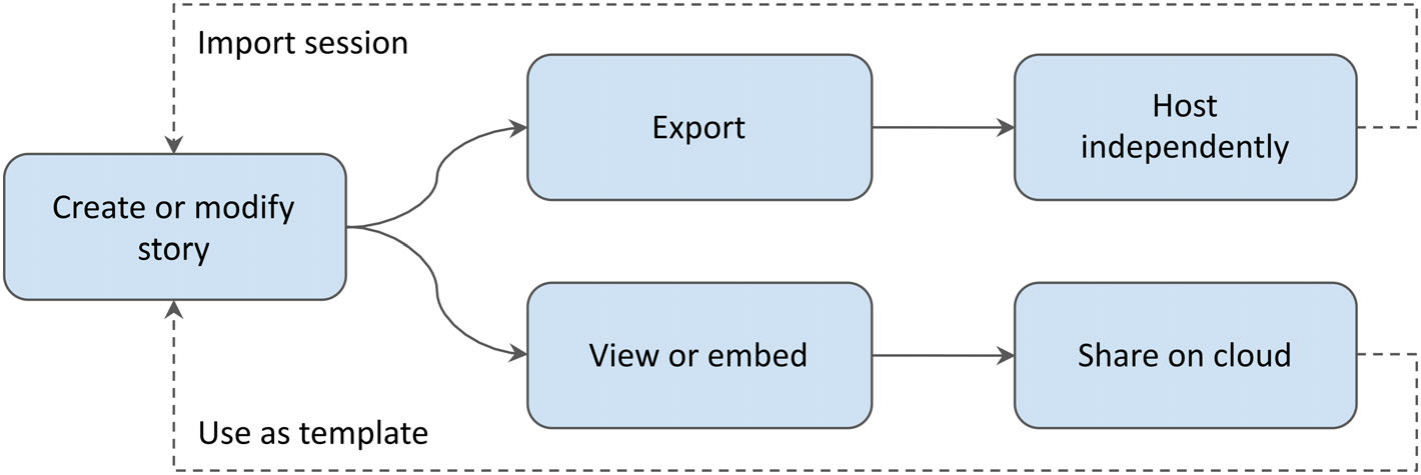
Sharing and forking. Users can share molecular stories either by publishing through an integrated cloud‐based service or by exporting a self‐contained package that can be hosted independently. In both cases, shared stories can be imported, edited, or used as templates to create derived narratives, supporting collaboration and reuse.

*Sharing and forking*. Stories can be published online, exported for local use, or packaged as standalone archives that remain fully functional without external dependencies. Each published story is automatically assigned a persistent URL, ensuring long‐term accessibility and reproducibility. Stories are also forkable, allowing users to create modified or extended versions for collaborative development. This functionality enables straightforward integration into web pages and online publications. Figure 3 illustrates the sharing and reuse workflow.

*Immersive visualization*. Support for augmented and virtual reality (AR/VR) provides stereoscopic rendering for enhanced spatial perception and engaging exploration in both research and education. Immersive features are accessible through any WebXR‐compatible browser and standard VR/AR hardware and require no additional software installation.

### 2.2 Architecture and implementation

MolViewStories is built as a modular, web‐based platform that integrates molecular visualization, interactive story authoring, and data management into a single, coherent environment. The system architecture defines the interaction between user interfaces, visualization components, and backend services, while the implementation leverages modern web technologies to ensure performance, scalability, and long‐term maintainability.

#### 2.2.1 Architecture

**FIGURE 4:**
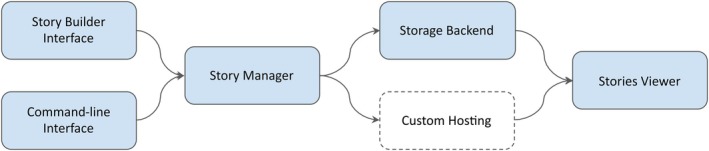
MolViewStories high‐level architecture diagram. MolViewStories architecture integrates user interfaces, management services, storage, and delivery components into a unified workflow. The Story Builder Interface and Command‐Line Interface (CLI) serve as authoring tools that generate and modify stories. These are managed by the Story Manager, which handles versioning, sharing, and publishing. Stories and related assets are stored in the Storage Backend, retrievable by the Stories Viewer for interactive display. Alternatively, stories can be exported to Custom Hosting environments, supporting independent deployment and long‐term accessibility independent from the MolViewStories infrastructure.

The MolViewStories architecture separates the user‐facing interfaces from the backend infrastructure to ensure scalability, reproducibility, and extensibility. Its design supports both interactive use and programmatic automation, enabling smooth transitions between graphical and command‐line workflows. The main components of the system are summarized below (see Figure 4).

*Story manager*. A component that organizes user sessions and shared stories. It allows saving, retrieving, and distributing interactive stories, while also supporting collaboration through versioning and forkable content.

*Story builder interface*. A web‐based graphical interface for assembling molecular stories. Users can create scenes, define animations, and add annotations or audio commentary, with all specifications stored in MolViewSpec for reproducibility.

*Command‐line interface* (*CLI*). A programmatic interface that enables story creation, modification, and export directly from scripts with instant live‐reload functionality to ease the development process. The CLI offers users the ability to iterate on their story locally while getting all of the benefits of version control while using a text editor or IDE of choice. The output of the CLI can also be built and shared via the web application.

*Storage backend*. The solution includes a persistent cloud storage service that maintains session data and shared stories to ensure reproducibility, long‐term availability, and compatibility of interactive visualizations across platforms. In addition to cloud‐based storage managed through the MolViewStories service, stories can also be exported and hosted completely independently, allowing users or institutions to deploy and serve interactive content on their own infrastructure without relying on external dependencies. This flexibility supports both centralized sharing and decentralized, self‐contained distribution of the molecular stories.

*Stories viewer*. The rendering component is built on top of the Mol* Viewer. It interprets MolViewSpec definitions to produce interactive 3D scenes, supports animations, and provides an immersive AR/VR experience.

The following section outlines how these components are realized in practice, describing the underlying technologies, frameworks, and implementation strategies that enable the functionality and performance of MolViewStories.

#### 2.2.2 Implementation

The platform is implemented using the TypeScript and Python programming languages with a modern web technology stack that supports reproducible molecular scene specification, high‐performance rendering, and robust backend storage. Each subsystem, ranging from the MolViewSpec‐based visualization layer to the storage and authentication services, operates as an independent module, while together they form an integrated framework for creating and sharing molecular stories. The key implementation aspects are outlined below:

*Scene specification with MolViewSpec*. Molecular scenes and animations are created by writing a script following MolViewSpec, a structured, machine‐readable format designed for reproducible molecular visualization. For MolViewStories, the specification is extended to capture both static states and time‐dependent animations, providing a powerful tool for molecular storytelling.

*Visualization engine*. The rendering layer is based on the Mol* Viewer, which provides robust support for structural biology data. Graphics are rendered using WebGL (https://www.khronos.org/registry/webgl/specs/latest/), allowing efficient 3D visualization in standard web browsers without plugins. Immersive modes are enabled through WebXR (https://immersiveweb.dev/), extending support to augmented and virtual reality headsets for stereoscopic exploration of molecular structures.

*User interface*. The web‐based user interface is implemented with Next.js (https://nextjs.org) and React (https://react.dev), providing a modern, component‐driven framework for building interactive molecular stories. This ensures responsive design, modularity, and extensibility across devices.

*Command‐line interface* (*CLI*). The CLI utilizes the Deno (https://deno.com/) runtime environment which enables TypeScript applications to be fully compiled and distributed as a single‐file binary application.

*Authentication*. Integration with LifeScience AAI Login (Linden et al., 2018) provides federated authentication, allowing secure access with institutional login or via personal accounts on GitHub, ORCID, Google, etc.

*Data storage*. Logged‐in users can store their sessions and share their stories (up to 100). Persistent storage of session data and shared stories is handled via a Flask (https://github.com/pallets/flask) backend and MinIO (https://github.com/minio/minio), an object storage system compatible with the Amazon S3 API. This architecture ensures scalability and long‐term data availability.

*Deployment and infrastructure*. The platform is developed and versioned on GitHub, with containerized services orchestrated using Kubernetes (https://kubernetes.io/) and deployed on the e‐INFRA CZ research infrastructure (https://www.e-infra.cz/). The architecture is modular, and the API can be deployed independently, allowing integration into external workflows or customized environments.

#### 2.2.3 Availability

The MolViewStories user interface is freely accessible at https://molstar.org/mol-view-stories alongside its documentation at https://molstar.org/mol-view-stories/docs. The complete source code is openly available under the MIT license at https://github.com/molstar/mol-view-stories.

## 3 RESULTS AND DISCUSSION

**FIGURE 5:**
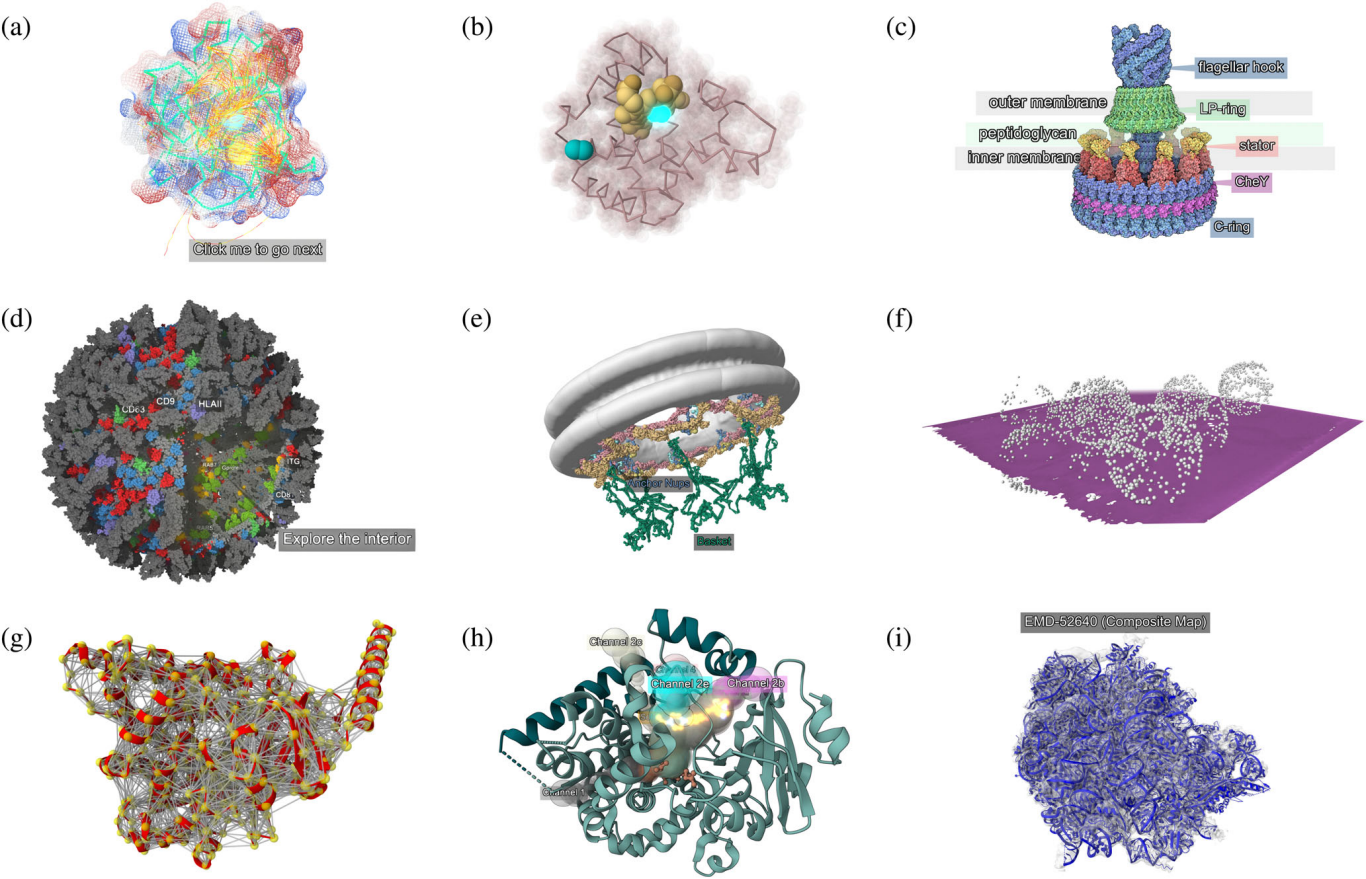
MolViewStories narratives demonstrating the range of interactive molecular storytelling. (a) Terms of Entrapment: the 1985 Cu,Zn superoxide dismutase animation remastered for real‐time, user‐driven exploration. (b) The Myoglobin Story: the first Molecule of the Month, authored by David Goodsell, reimagined as an interactive narrative with animations and audio commentary. (c) The Flagellar Motor Story: the 300th Molecule of the Month, the last entry authored by David Goodsell, reimagined as an interactive narrative with animations and audio commentary. (d) The Exosome Story: an immersive mesoscale visualization integrating guided narration and dynamic highlighting of structural features. (e) NPC Basket: a multi‐scale integrative model of the nuclear pore complex basket illustrating structural organization and transport dynamics. (f) Subtomogram Averages: linking cryo‐ET data from whole tomogram context to atomic‐level structures. (g) How AlphaFind Works: explaining large‐scale structural similarity searches using vector embeddings across protein space. (h) P450 Cytochromes with Channels: visualizing substrate access, inhibitor binding, and channel networks in CYP3A4. (i) Composite Maps in EMDB: an interactive explanation of composite map deposition and relationships among refinement and consensus maps. All examples are publicly available on the MolViewStories landing page at https://molstar.org/mol‐view‐stories and can be used as templates for building new narratives.

We demonstrate MolViewStories using a diverse set of molecular narratives that showcase its flexibility across multiple biological contexts and visualization scales (see Figure 5). Rather than presenting a curated selection of “best” examples, these stories intentionally span a range of goals, from historical reinterpretation and educational exposition to integrative structural modeling and computational analysis. Together, they illustrate how MolViewStories can accommodate different levels of narrative depth, scientific focus, and visual complexity, depending on author intent and audience. Each example emphasizes distinct platform capabilities such as animation, audio narration, multi‐scale visualization, or integration with structural biology resources, and all can be reused or adapted as templates for new narratives. Collectively, these stories demonstrate how MolViewStories enables clear, reproducible, and accessible communication of molecular structures across research, education, and outreach contexts.

### 3.1 Terms of entrapment/Back to the future story (1985's animation remastered)

The short film *Terms of Entrapment* (https://www.youtube.com/watch?v=Jk7-CB10-yY) was a landmark in molecular graphics, recognized at the SIGGRAPH 1985 Film and Video show (Olson et al., 1985) for its pioneering use of real‐time computer graphics to depict the structure and function of copper–zinc superoxide dismutase (SOD) at atomic resolution. Based on the x‐ray crystallographic structure of bovine Cu,Zn SOD (PDB ID: 2SOD) (Getzoff et al., 1983), it visualized in unprecedented detail how the enzyme's Greek‐key β‐barrel fold, dimeric interface, and electrostatic potential contribute to its stability and catalytic efficiency in detoxifying superoxide radicals. In MolViewStories, this classic animation has been faithfully re‐created and extended into an interactive experience, allowing real‐time, user‐driven exploration that was technologically impossible in 1984. The original audio tape describing the movie was also digitized and synchronized with the visualization to preserve its historical context.

#### 3.1.1 1st molecule of the month

The *Myoglobin Story* is based on the very first Molecule of the Month^2025^, published by David Goodsell in January 2000 (Iwasa et al., 2025), which launched what has since become an iconic educational series. To commemorate this milestone, we reimagined the original Myoglobin entry as an interactive narrative. The story is divided into three parts covering the first protein structure determination, the biology of myoglobin in whales, and the principles of oxygen binding and protein dynamics. It features interactive animations, descriptive text, and synchronized audio commentary generated by AI voice (https://evernote.com/ai-text-to-voice), allowing users to explore PDB structures such as PDB ID 1MBN (Kendrew et al., 1960) directly in the browser. A link to this interactive version has been added to the corresponding Molecule of the Month page.

#### 3.1.2 300th molecule of the month

The *Flagellar Motor Story* is based on the 300th and last Molecule of the Month^2025^ authored by David Goodsell before his retirement, concluding 25 years of scientific storytelling through the Protein Data Bank's educational series (Iwasa et al., 2025). This interactive narrative explores the atomic and functional architecture of the bacterial flagellar motor, a remarkable rotary machine that propels bacterial motion. The story is structured into thematic segments that describe the organization of the motor, the molecular basis of rotation, directional switching, and stator function. Through coordinated animations, synchronized audio narration, and real‐time 3D exploration, the narrative closely integrates explanation with visualization to guide the viewer through complex mechanical concepts. While largely faithful to the original educational material, the interactive format highlights how tightly coupled narration and visual emphasis can enhance understanding and also illustrates opportunities for further refinement of legacy content within interactive storytelling frameworks.

#### 3.1.3 Exosome story

The *Exosome Story* was originally introduced as a guided tour in Mesoscale Explorer (https://molstar.org/me/viewer/?example=cellpack‐exosome‐tour&hide‐controls=1), providing one of the first narrative‐driven explorations of mesoscale models accessible to a broad audience. Exosomes are nanoscale vesicles secreted by most cell types into bodily fluids such as blood, saliva, and breast milk. Once thought to be mere *cellular garbage shuttles* (Raposo & Stoorvogel, 2013), they are now recognized as *information carriers* in intercellular communication (Huang‐Doran et al., 2017), with important implications for cancer diagnostics and therapy (Barile & Vassalli, 2017). The underlying 3D structural model of the exosome is described in Jiménez et al. (Jiménez et al., 2019) We ported this guided tour and enriched it with *descriptive audio* to bring the narrative to life, along with new interactive animations, including clipping planes and emissive illumination, that dynamically highlight proteins and structural features. By combining integrative modeling, interactive narration, and enhanced visualization, this work transforms static exosome models into an immersive molecular storytelling experience.

#### 3.1.4 NPC basket

This story illustrates the structure of the yeast nuclear pore complex (NPC) basket as determined by integrative modeling using cryo‐electron tomography and crosslinking data (Singh et al., 2024). The NPC, composed of over 500 proteins, mediates all molecular traffic between the nucleus and cytoplasm. The flexible, cage‐like basket on the nuclear side facilitates mRNA transport and chromatin organization. The story focuses on the overall architecture and integrative structural organization of the basket, using animations and annotations to describe how its suspension‐bridge‐like design is stabilized by anchored proteins connected to the nuclear ring, with coiled filaments extending into the nucleoplasm. Visualization of coarse‐grained and Gaussian surface representations provides insight into the basket's modular organization, flexibility, and transport dynamics, illustrating the potential of MolViewStories for multi‐scale visualization of integrative structures. As illustrated by the other examples, the platform also provides the capability to incorporate additional biological detail, such as conformational variability or intrinsic disorder, further enriching the depiction of NPC function.

#### 3.1.5 Subtomogram averages

This story presents atomic models derived from subtomogram average volumetric data within its associated tomographic data, linking large‐scale macromolecular organization to atomic detail. It begins with a low‐resolution overview of an entire tomogram, where particles (macromolecules) are identified as spheres to indicate macromolecular spatial distribution, and then zooms into a single particle for a detailed view. Finally, a tomogram slice is displayed, superimposing a subtomogram average and the corresponding atomic model. Averaging is essential because single subtomograms extracted from tomograms have a low signal‐to‐noise ratio due to the limited electron dose used to image the specimen without destroying it. By aligning and averaging hundreds or more subtomograms of the same particle, random noise averages out, and the structural signal is amplified, significantly improving the signal‐to‐noise ratio. This enhancement can resolve sufficient detail for atomic‐level interpretation, which would be impossible from single subtomograms alone (Wan & Briggs, 2016). Particle positions and orientations were derived from a RELION (Bharat et al., 2015; Scheres, 2012) star file and used to position each subtomogram within the tomogram volume. The atomic model fitted to a subtomogram average represents a gag protein fragment from HIV‐1 (Schur et al., 2016). The story demonstrates how cryogenic‐sample electron tomography (cryoET) data can be explored interactively, highlighting the relationships of large‐scale macromolecular and cellular‐scale organization to molecular‐ and atomic‐scale structures.

#### 3.1.6 How AlphaFind works

This story demonstrates large‐scale protein structural similarity search using the Cytochrome P450 enzyme family as a representative example. It begins with an animation of a 3D alignment between two P450 structures, showing how similarity metrics such as the TM‐score (Zhang & Skolnick, 2004) quantify structural relationships. The narrative then shifts to explain how such comparisons are scaled computationally. The Cytochrome P450 from maize (https://www.uniprot.org/uniprotkb/A0A1D6JW22) is visualized as a molecular graph in which nodes exchange information through message passing, generating a compact vector embedding that captures key spatial and chemical features of the protein. The story then transitions into an interactive depiction of this embedding space, illustrating how structural neighbors are identified rapidly through proximity in the learned representation. This approach underlies tools such as AlphaFind (Procházka et al., 2024), which use vector embeddings to efficiently screen more than 200 million structures from the AlphaFold Protein Structure Database (Varadi, Anyango, et al., 2022), quickly narrowing the search to the most relevant candidates for detailed structural alignment.

#### 3.1.7 P450 cytochromes with channels

This story focuses on the structural features of human cytochrome P450 3A4 (CYP3A4), a key enzyme responsible for metabolizing a significant proportion of clinically used drugs (Zanger & Schwab, 2013). The narrative examines the enzyme's deeply buried active site, defined by the heme catalytic center and a flexible region containing a cluster of phenylalanine residues crucial for substrate recognition and binding (Williams et al., 2004). The story presents the visualizations of substrate access channels that connect this active site to the external environment. These channels were identified using the computational tool MOLEonline (Pravda et al., 2018). The story illustrates how inhibitors like ketoconazole bind within these channels to block substrate access and modulate enzyme activity (Ekroos & Sjögren, 2006).

#### 3.1.8 Composite maps in EMDB

Composite map deposition remains a common source of confusion for contributors to the Electron Microscopy Data Bank (EMDB). According to the community recommendations established in 2020 (Kleywegt et al., 2024), composite map submissions must include, as distinct entries, the composite map, the constituent focused refinement maps, and a corresponding consensus or full map. This format allows separate validation while maintaining explicit cross‐references between entries. To aid understanding, the EMDB now provides a Composite Map Deposition Guide (https://www.ebi.ac.uk/emdb/pages/documentation/deposition/composite_map). With MolViewStories, this process is presented interactively, showing how composite maps relate to their focused and consensus counterparts, helping depositors and reviewers visualize complex map hierarchies.

#### 3.1.9 MolViewSpec feature tutorial

This tutorial story serves as a guided walkthrough of MolViewSpec features and syntax. Each component of the specification is illustrated with a minimal working example, accompanied by JavaScript code snippets and direct links to the documentation. It provides new users with a structured introduction to authoring molecular stories, showcasing how MolViewSpec can capture both static scenes and dynamic animations reproducibly.

### 3.2 Future work

The development of MolViewStories presented in this work establishes a solid technical and conceptual foundation for interactive molecular storytelling. Building on this framework, future efforts will focus on extending accessibility, automation, and integration with existing scientific resources to enhance both research and educational impact. The directions outlined below highlight ongoing and planned initiatives aimed at broadening the platform's functionality and reach.

*AI and LLMs*. The rapid advancement of generative AI and conversational systems is transforming how scientific content is created and shared. Within MolViewStories, we have already experimented with tools such as DeepWiki, ChatGPT, and Evernote to assist in drafting narrative text and generating illustrative examples. Looking ahead, integration of AI capabilities could further streamline story creation and interaction. The declarative structure of the MolViewSpec API lends itself naturally to code completion and natural‐language–based scene generation, while dynamic markdown and dialogue‐driven interfaces could enable users to request contextual explanations or visual adjustments in real time. Although current AI‐generated molecular animations remain limited in accuracy, the curated and structured examples produced through MolViewStories can serve as valuable training resources for future systems, fostering the development of more reliable, data‐driven molecular visualization methods.

*UI‐driven builder*. The current implementation requires users to construct stories via code. A fully UI‐driven builder is planned to lower the entry barrier, making story creation more accessible to a broader user base, including those without programming expertise. In particular, interactive UI elements and gizmo helpers will greatly assist in designing camera paths and protein motions.

*Integration with structural biology resources*. Future work will also focus on integration with major structural biology repositories. In collaboration with PDBe and RCSB PDB, MolViewStories could be incorporated into deposition systems and structure pages, providing interactive visualizations alongside deposited data.

*Educational and outreach content*. Ongoing collaboration with PDBe and RCSB PDB aims to extend the Molecule of the Month series with immersive, interactive narratives. By combining guided storytelling with augmented and virtual reality (AR/VR) visualization, these materials will make molecular structures more engaging and intuitive, supporting classroom teaching, public exhibitions, and broader science outreach.

## 4 CONCLUSION

MolViewStories provides an open, accessible, and extensible platform for creating and sharing interactive molecular visualizations. By combining a graphical and code‐based story builder with a robust visualization engine, it enables researchers, educators, and students to move beyond static figures and engage with molecular data through dynamic, narrative‐driven presentations. The platform supports reproducibility through the MolViewSpec format, offers flexible interfaces for both exploratory and automated use, and facilitates long‐term sharing through standalone, web‐compatible packages.

The example stories presented here demonstrate how complex structural data can be transformed into clear, engaging, and reusable narratives for research, education, and outreach. Looking forward, continued integration with community databases and expansion of authoring tools will further lower barriers to participation and sustain the growth of interactive molecular storytelling.

Together, these developments position MolViewStories as a foundation for open, transparent, and accessible communication of structural biology data.

## AUTHOR CONTRIBUTIONS

**Terézia Slanináková:** Data curation; formal analysis; software; visualization; writing – original draft; writing – review and editing. **Zachary Charlop‐Powers:** Conceptualization; data curation; formal analysis; software; visualization; writing – original draft; writing – review and editing. **Viktoriia Doshchenko:** Data curation; software; visualization; writing – original draft; writing – review and editing. **Alexander S. Rose:** Software; writing – review and editing. **Adam Midlik:** Data curation; software; writing – original draft; writing – review and editing. **Anna Sekuła:** Data curation; software; writing – original draft; writing – review and editing. **Neli Fonseca:** Data curation; software; writing – original draft; writing – review and editing. **Kyle L. Morris:** Resources; writing – review and editing. **Stephen K. Burley:** Resources; writing – review and editing. **Sameer Velankar:** Resources; writing – review and editing. **Jennifer Fleming:** Project administration; data curation; resources; writing – original draft; writing – review and editing. **Brinda Vallat:** Data curation; writing – original draft; writing – review and editing. **Ludovic Autin:** Data curation; software; visualization; writing – original draft; writing – review and editing. **David Sehnal:** Conceptualization; formal analysis; investigation; methodology; software; supervision; funding acquisition; resources; validation; visualization; writing – original draft; writing – review and editing.

## CONFLICT OF INTEREST STATEMENT

The authors declare no conflicts of interest.

## Data Availability

The data that support the findings of this study are openly available in figshare at http://doi.org/10.6084/m9.figshare.30621254, reference number 10.6084/m9.figshare.30621254.
